# Natural Iron Oxide Nanoparticles Produced by Aquatic Magnetotactic Bacteria as Ideal Nanozymes for Nano-Guided Biosensing Platforms—A Systematic Review

**DOI:** 10.3390/bios15090590

**Published:** 2025-09-08

**Authors:** Natalia Lorela Paul, Catalin Ovidiu Popa, Rodica Elena Ionescu

**Affiliations:** 1 Materials Science and Engineering Department, Faculty of Materials and Environmental Engineering, Technical University of Cluj-Napoca, 400641 Cluj-Napoca, Romania; natalia.paul@utt.fr (N.L.P.); catalin.popa@stm.utcluj.ro (C.O.P.); 2 Light, Nanomaterials and Nanotechnology (L2n) Laboratory, CNRS UMR 7076, University of Technology of Troyes, 12 Rue Marie Curie, CS 42060, CEDEX, 10004 Troyes, France; 3 Eut+ Institute for Nanomaterials & Nanotechnologies EUTINN, European University of Technology, European Union

**Keywords:** natural iron oxide, nanozymes, nanocatalysts, magnetotactic bacteria, nano-guided biosensors

## Abstract

In response to the ongoing challenges associated with natural enzymes, their high production costs, low stability and limited functionality; nanozymes have rapidly emerged as versatile alternative. Such nanocatalysts, based on nanomaterials and nanostructures, offer remarkable tunability of physicochemical properties and excellent durability, and adapt themselves effectively to the requirements of modern biotechnological applications. This review article aims to provide a comprehensive overview of recent advances in the use of naturally occurring iron oxide nanoparticles, produced by magnetotactic bacteria, and to highlight their emerging role as key elements in the development of the new generation of nano-guided biosensors. It provides a comprehensive and systematic analysis of publications in the Web of Science database between 2022 and August 2025, conducted in accordance with PRISMA guidelines. The aim was to assess the current state of the art and identify knowledge gaps in the exploration and application of magnetotactic bacteria as natural and sustainable sources in the design of next-generation biosensors. The natural nanoparticles, formed through biological processes, represent a unique and sustainable alternative to synthetic nanoparticles, offering naturally mimetic enzymatic activity, high biocompatibility, and exceptional stability. This approach opens up revolutionary perspectives in the field of biosensors, proposing a new class of functional materials, iron nanoparticles of biological origin, capable of fundamentally changing the performance, sustainability and reliability of future nanoenzymatic sensing platforms.

## 1. Introduction

With the advances and expansions in nanotechnology, biomaterials science and technological engineering, the field of nanozymes has seen remarkable progress. Nanozymes are smart alternatives to natural enzymes, with many key properties: increased stability, lower costs, the possibility of being modified and shaped, as well as reduced difficulties in the manufacturing process [1,2,3]. They are inorganic materials or a combination of organic–inorganic materials with the ability to catalyze specific reactions, similar to natural enzymes. The major difference, compared to the latter, is that nanozymes are not susceptible to denaturation under extreme conditions (temperature, pH, organic solvents),which gives them a significant advantage in being used in numerous applications in various fields of applicability (biomarker detection, neurological diseases, nanotherapy, biomedical applications, environmental protection, and agricultural production) [3,4,5,6,7].

**Figure 1:**
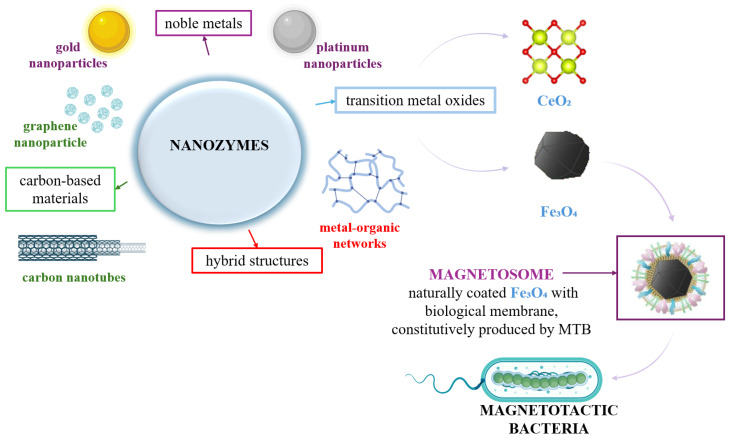
Various types of nanozymes including natural iron oxide nanoparticles; CeO_2_—cerium oxide, Fe_3_O_4_—iron oxide.

Since the first publication (2007) on the ability of iron oxide nanoparticles to mimic the enzymatic activity of peroxidase [8], research into these enzymes has expanded significantly. However, this only happened after 2015, when the field of nanozymes began to gain momentum and visibility, thanks to decisive scientific articles and improvements of methods of synthesis, characterization and functionalization of materials at the nanoscale. This period of development coincided with a growing need to replace natural enzymes in medical, industrial and environmental applications, as these enzymes, despite being highly efficient and selective, tend to be unstable under extreme pH and temperature conditions, sensitive to denaturation, and expensive in terms of isolation, purification and preservation. In this context, nanozymes offer a promising alternative, thanks to their remarkable physicochemical properties, excellent stability and numerous possibilities for structural and functional modification. These studies have facilitated the development of several types of nanozymes. Among the most studied mimetic enzymatic activities are those with catalytic activities such as catalase (CAT) [9], oxidase [10] and superoxide dismutase (SOD) [11]. Furthermore, through the use of a variety of nanomaterials, includes transition metal oxides (Fe_3_O_4_ or CeO_2_) [12], noble metals (gold or platinum) [13], carbon-based materials (graphene or carbon nanotubes) [14], but also hybrid structures (metal–organic networks) [15] (Figure 1).

Nanozymes can be used in a wide range of applications, from medical diagnostics through to electrochemical biosensors for the detection of tumor markers, glucose or pathogens, to advanced oncological therapies, in which nanozymes are used to generate reactive oxygen species (ROS) in the tumor microenvironment, thereby contributing to the selective destruction of cancer cells [16]. In addition, they prove their effectiveness in environmental remediation, contributing to the breakdown of organic pollutants and the neutralization of toxic compounds, as well as in biotechnology, where they can replace natural enzymes in catalytic processes [17]. However, challenges remain, particularly with regard to substrate non-specificity, catalytic mechanisms that are not fully understood in some cases, limited biocompatibility of certain materials, and toxicological potential in biomedical applications [16]. Therefore, these limitations raise a number of questions: Do natural nano-based materials have intrinsic properties similar to enzymes? If so, how do they behave in terms of biocompatibility? Can they overcome at least some of the limitations currently found in nanomaterials used as nanozymes?

In this context, this review presents the inherent potential of the use of iron oxide nanoparticles produced by magnetotactic bacteria (MTB) (Figure 1). Even though the use of iron oxide nanoparticles for their nanoenzyme properties is frequently addressed in the literature, the use of natural NPs derived from magnetotactic bacteria is not yet addressed as such. We support this view by pointing the research on magnetotactic bacteria and the iron oxide nanoparticles they mineralize primarily on their use as versatile analytical tools, particularly in medical applications (hyperthermia [18,19,20], drug delivery [21,22,23], contrast agents [24,25], cancer treatment [18,26,27], and enzyme immobilization [22,28]) and environmental applications (heavy metal capture and organic pollutants from wastewater and sediments [29,30], pathogen capture, and food safety application [31,32,33]). Thus, up to the present time, we have not identified in the literature the addressing of iron oxide NP from MTB as natural nanozymes, especially in the construction of biosensors, either alone or in combination with other types of nanozymes. Consequently, we aimed to address this gap in the literature in order to provide a theoretical and summarized support for the possibility of using these nanoparticles in the construction of versatile biosensors.

MTBs are a special group of Gram-negative prokaryotic microorganisms with several unique characteristics that can revolutionize various fields of activity [34]. One of the most notable features of MTB is the fact that they possess magnetosomes. These magnetosomes are regularly produced in the MTB membrane by converting environmental iron sources (Fe^2+^/Fe^3+^) and mineralizing them into nanometric particles of magnetite (Fe_3_O_4_) or greigite (Fe_3_S_4_) nanoparticles [23]. Another feature is the biocompatibility of MTB mineralized nanoparticles, which is a result of their immediate adaptation to natural biological membranes. This feature alone overcomes many obstacles faced by the scientific community in its attempts to improve the properties of nanomaterials used in biomedical applications [34].

In vitro and in vivo studies highlight notable differences between synthetic iron oxide nanoparticles and those naturally produced by magnetotactic bacteria [35,36,37,38]. Synthetic Fe_3_O_4_ nanoparticles, although exhibiting high catalytic activity, may exhibit adverse effects due to heterogeneous surface, non-uniform size distribution and possible impurities originating from chemical synthesis methods, which can induce oxidative stress and cytotoxicity at the cellular level [35,39]. In contrast, MTB magnetosomes are characterized by a uniform morphology, a natural phospholipid surface and intrinsic biocompatibility, demonstrating low toxicity and good tolerance in animal models. Furthermore, in complex biological environments, magnetosomes exhibit superior stability and more efficient physiological clearance compared to synthetic nanoparticles, which reduces the risk of long-term tissue accumulation [40,41,42]. This combination of properties makes them attractive candidates for biosensing applications, where both biological safety and signal reproducibility are essential. The use of magnetosomes may allow for more stable and selective detection platforms with increased compatibility for biological interfaces, thus offering a clear advantage over synthetic analogues.

In order to examine the possibility of using these natural nanoparticles as nanozymes, it is necessary to gain a thorough understanding of the methods of characterizing iron oxide and the principles of its catalytic action. It is also necessary to understand the formation of these magnetic nanoparticles by MTB, their extraction from these bacteria, as well as the methods of characterizing and evaluating them.

## 2. Information from Recent Bibliographic Studies

### 2.1. Data Collection

In order to obtain a comprehensive picture of the progress made to date in the field of nanozymes, a review of the scientific literature was conducted.

In the first stage, a Web of Science database was compiled (accessed on 16 August 2025), which contains most of the resources related to this topic. It includes both review and early access documents, as well as book chapters or associated data, but above all, it includes recent documents on the latest advances in the field of nanozymes with the option to focus the search to best suit specific preferences.

**Figure 2:**
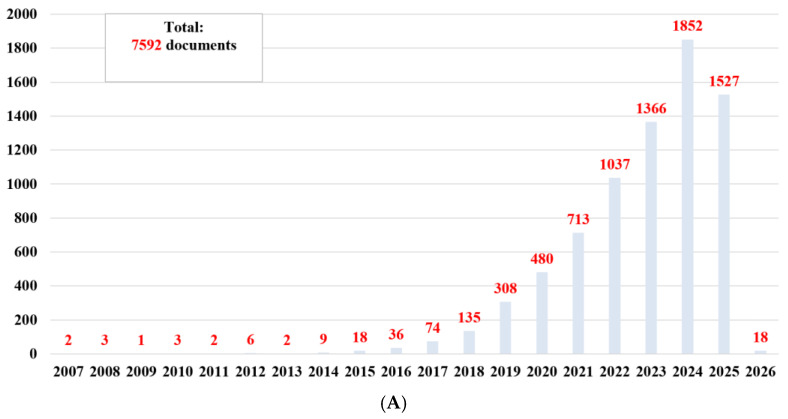

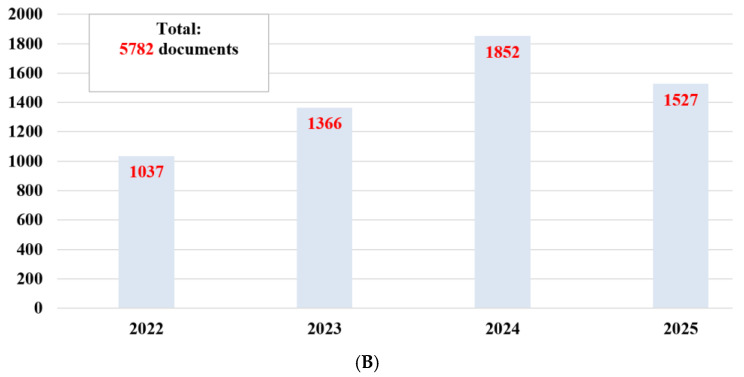
Number of publications related to the topic “nanozyme” in the Web Of Science database since their discovery in 2007 (**A**) and the increased number of publications between 2022 and August 2025 (**B**).

Thus, 7592 documents published in the database since the discovery of nanozymes (in 2007) (Figure 2A) were obtained. If we look at the total number of publications (Figure 2), it can be said that there is considerable interest in basic research and applications in this field. However, if we examine the distribution of the number of publications by year of publication, especially from 2022 (5782 documents) (Figure 2B), we are seeing exponential growth year after year, reflecting both the general interest and the need for research based on nanozymes.

### 2.2. Data Analysis

In the second stage, the database query was refined to include documents published between 2022 and August 2025. In addition, the search was refined by document type (review articles and open access documents), and language of publication (documents in English). This query yielded a total of 183 articles.

**Figure 3:**
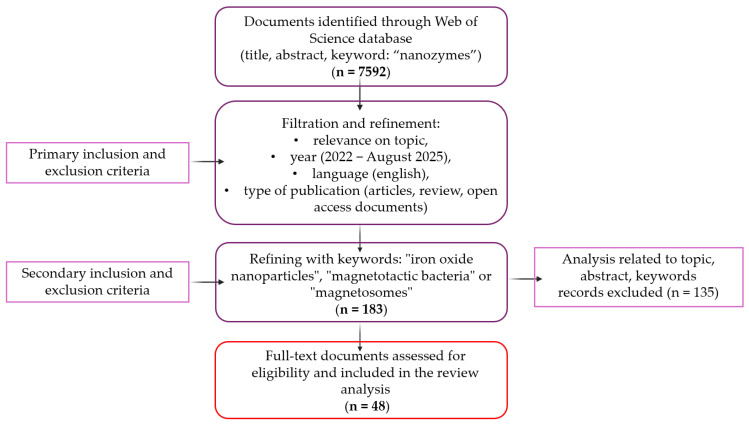
Methodology of research using PRISMA-style flow diagram detailing keywords related to nanozymes, iron oxide nanoparticles, magnetotactic bacteria or magnetosomes for the period 2022–August 2025.

At the last stage, the refinement included a search focused on the specific research that is the topic review, more precisely a combination of key terms (“nanozyme”, “iron oxide nanoparticles”, “magnetotactic bacteria” or “magnetosomes”). This refinement resulted in the selection of 48 articles (articles and review articles) and were selected for a detailed analysis of the current state of progress in the field of nanozymes, as well as the characterization of iron oxide and the analysis of their enzyme-mimicking activities. Both the review of documents identified and their classification into categories were carried out following PRISMA guidelines, Figure 3.

As can be seen in Figure 3, the primary inclusion criteria were based on relevance and concordance with the topic of this review (the field of nanozymes), the publication period of the documents (2022–August 2025), the language of publication (English), and the type of document (open access, articles and review articles). As for the secondary inclusion criteria, they aimed at concordance with the specific details on which this review is based, more precisely on the identification of documents correlated with magnetotactic bacteria. This was achieved by using the combination of keywords (“iron oxide nanoparticles”, “magnetotactic bacteria”, “magnetosomes”). The exclusion of documents (out of the total of 183 obtained) was based on the analysis of relevance to the central topic through the analysis of the abstract and keywords; thus, 135 publications were excluded, and 48 were subsequently analyzed in detail.

**Figure 4:**
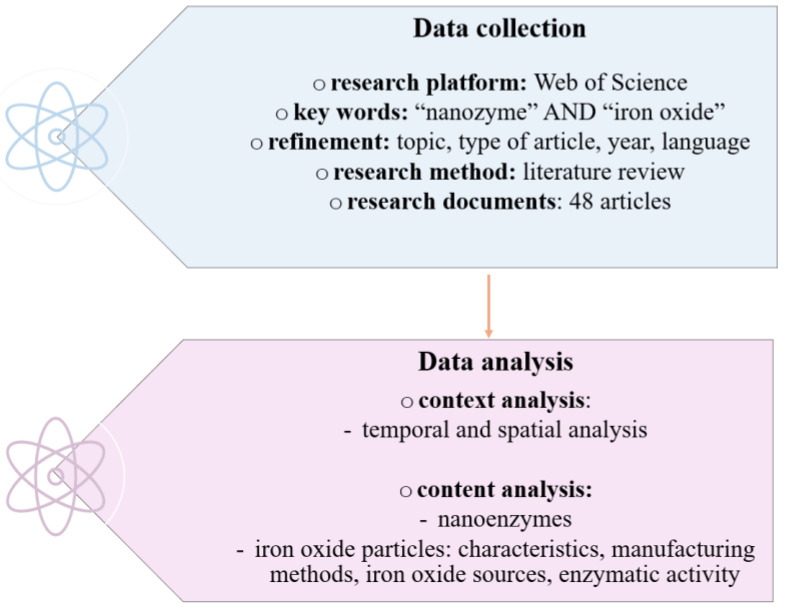
Research methodology regarding two topics: nanozymes and iron oxide.

After the in-depth analysis, information regarding the progress made in the field of nanozymes, the properties of iron oxide particles, the characteristics and methods for obtaining the latter, were structured into context and content elements (Figure 4).

*Context elements* were made in close correlation with temporal and spatial aspects in order to reveal the growing interest in this developing field, which is constantly being researched. On the other hand, *content elements* were arranged around the element of interest in this context represented by nanozymes and iron oxide particles, as well as the elements that define them: characteristics, production methods, sources of iron oxide and their enzymatic activity.

#### 2.2.1. *Context Analysis*

The investigation of the catalytic properties of various substances has contributed to the accelerated development of the field of nanozymes, which dates back almost two decades. The concerns, curiosity and intense interest of researchers in recent years have facilitated the development of a wide range of substances that have enzyme-like activity such as nanozymes.

**Figure 5:**
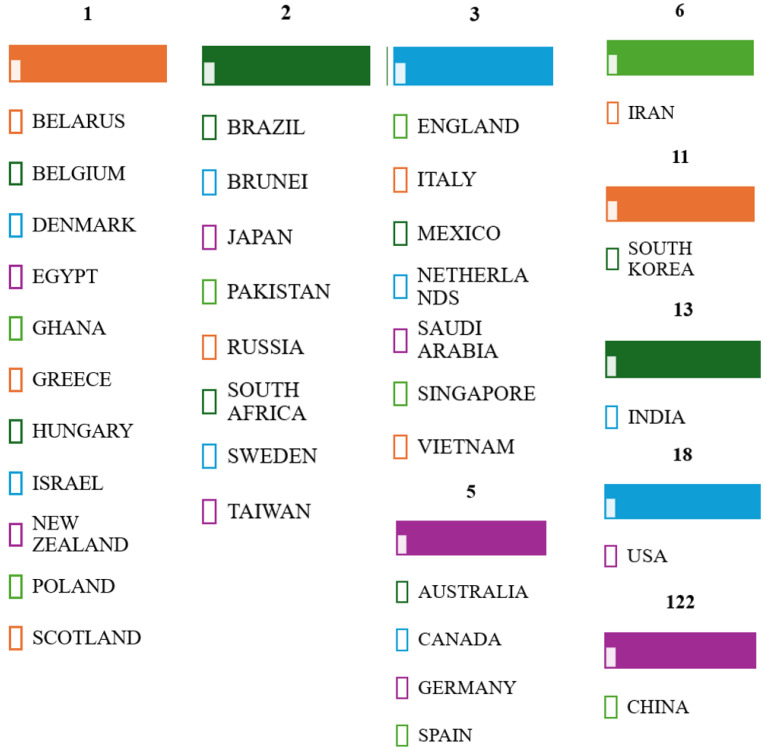
Global spatial distribution of the number of publications related to the use of iron oxide as nanozyme according to the Web of Science database collected between 2022 and August 2025.

Contextual analysis focused on temporal and spatial landmarks from the identified sources. The studies published between 2022 and June 2025 (Figure 2B) indicate ongoing efforts in the research, development, and improvement of catalytically active materials, as well as a constant interest in the impact and documentation of sustainable methods to contribute to the development of this field. Furthermore, Figure 5 reports on the global research distribution, according to the Web of Science database, in the direction of nanozymes and the interest in using the catalytic properties of iron oxide.

#### 2.2.2. *Content Analysis*

Although the term nanoenzyme was first introduced in 2007, interest in this field has only grown significantly over the past decade. This is due to a combination of factors that mark a turning point in the field of research. These factors are specifically directed towards advances in nanotechnology, the controlled synthesis of nanoparticles and nanoscale materials, their advanced characterization, and the detailed analysis and understanding of the catalytic mechanisms of nanoscale particles. It is only in light of the growing interdisciplinary understanding of nanotechnological mechanisms that the growth of the nanoenzymatic field has been possible.

The authors’ interests in this review lie in investigating the catalytic properties of iron oxide nanoparticles, their characterization, synthesis methods, iron oxide sources (natural and artificial), as well as their potential use as nanozymes. All of these are essential to be able to compare particles obtained by synthetic routes with those obtained constitutively by extraction from natural magnetotactic bacteria.

Even though numerous studies have been conducted in this direction, there is stillroom for numerous improvements, especially regarding their application in various fields, such as biosensors and biomedical applications.

## 3. Nanozymes: State of the Art

Over the past two decades, the field of nanozymes has seen accelerated progress, attracting great interest from the scientific community due to their properties similar to those of natural enzymes. Other aspects that have attracted significant attention are those related to low costs, superior stability (compared to natural enzymes), but above all, functional versatility.

Nanoparticles and nanoscale materials used as nanozymes offer viable alternatives to natural enzymes, as they are capable of mimicking various enzymatic activities and find applications in various fields (biomedical, industrial, environmental and agricultural) [12,13,20]. The applicability, specificity and efficiency of nanozymes are directly influenced by their structure, which is the fundamental component of their catalytic and functional properties. The remarkable architectural diversity of nanozymes results in the improved specificity and variability of structural features, respectively, through specific enzymatic activities.

### 3.1. Natural Enzymes vs. Nanozymes

What are enzymes? Why are they important? And why do we need alternative enzymes?

Enzymes are natural biocatalysts, essential in biological processes. They facilitate and accelerate biochemical reactions in the biological microenvironment, participate in numerous body processes (growth, digestion, development, DNA replication, protein synthesis, transport and mobilization of energy resources) and ensure the unfolding of biological processes and the maintenance of life [43].

Most enzymes are proteins, composed of chains of amino acids that fold into structures with different three-dimensional conformations, determining their catalytic function [21]. The role of enzymes in living organisms is enormous and vital, and they participate in all the processes and mechanisms that ensure their growth and development. In addition to this vital role, enzymes, which are powerful natural biocatalysts, have catalytic activity and high substrate specificity, and they find applications in varied fields such as biomedicine [22], food processing [23], chemistry and chemical engineering [24], environmental remediation [25] and bioengineering [26].

Despite their crucial roles, enzymes have disadvantages that limit their use in certain applications and fields. The most important of these are reduced stability to temperature or pH changes, difficulties in storage and reuse, and high production costs [44]. For example, their impressive catalytic efficiency, combined with their biocompatibility, specificity, and selectivity, make enzymes ideal catalysts for industrial applications. However, their practical application in industrial applications is often limited by difficulties related to reuse, separation, and stability [28]. Another aspect that limits the use of natural enzymes concerns the difficulties of functionalization and modification to adapt them to new substrates or reaction conditions [29].

**Figure 6:**
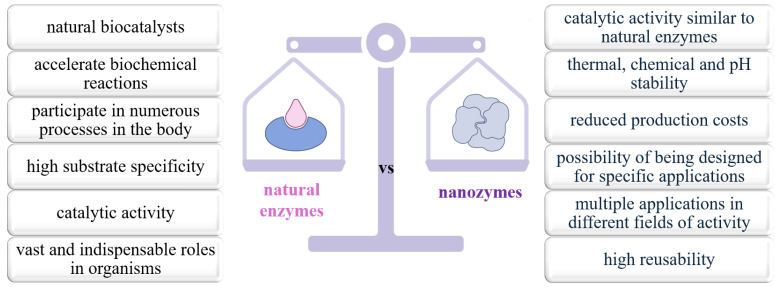
Benefits of using natural enzymes vs. nanozymes.

In this context, nanozymes, in the form of nanomaterials with catalytic activity similar to natural enzymes, have been developed in an attempt to overcome these limitations. Nanozymes offer advantages that address, in particular, the limitations of natural enzymes, namely thermal, chemical and pH stability, reduced production costs and the possibility of design for specific applications [30] (Figure 6). However, this does not mean that they are perfect or flawless, and challenges regarding substrate specificity or their toxicological potential have often been reported [31,32].

In Table 1, we summarize the main characteristics that define the advantages and disadvantages, potential properties as well as limitations of nanozymes and natural enzymes.

### 3.2. Factors Affecting the Activity of Nanozymes

**Figure 7:**
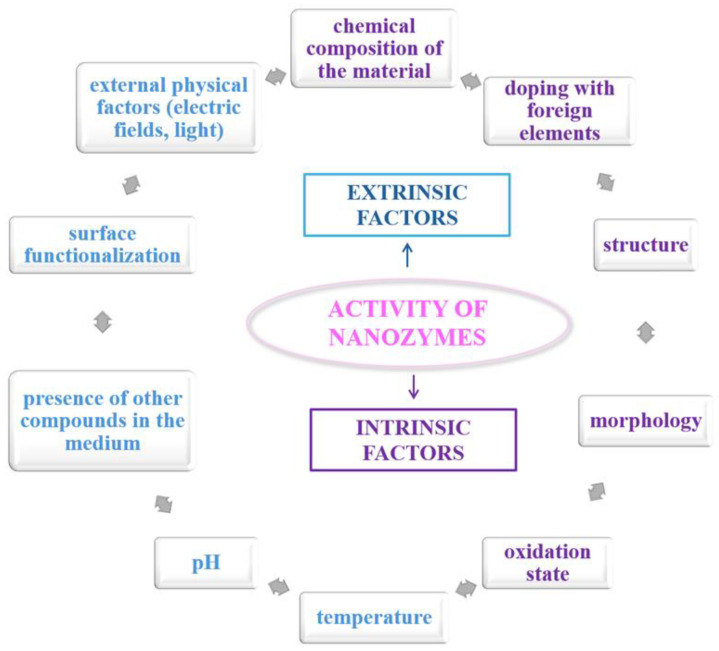
Factors influencing the activity of nanozymes.

The emerging class of nanozymes is a class of nanostructured materials that closely mimic the catalytic functions of natural enzymes and that attempt to overcome the barriers of natural enzymes by bringing improvements in the areas of structural stability, chemical durability, and functional adaptability [61]. The catalytic activity of these nanozymes is not a property defined by ideal or fixed parameters, rather, it depends on various of factors (Figure 7) that ultimately define their efficiency, specificity, but also their applicability in different contexts, domains or industries. In order to design rational, manageable and efficient nanozymes, it is necessary to know these factors, as well as to understand how they can affect their catalytic activity [62].

One of the most important intrinsic factors is the chemical composition of the material used. Whether it is a nanostructured compound or a metal or metal oxide, the type and composition of this material will further determine the ability of the nanoenzyme to catalyze certain types of reactions. For example, cerium oxide nanoparticles can be used to mimic the activity of CAT and SOD (due to their ability to switch between Ce^3+^ and Ce^4+^ oxidation states) [46]. Conversely, iron nanoparticles have shown similar properties, but in terms of mimicking peroxidase activity [47]. This redox versatility is essential for the functionality of nanozymes in variable biological environments [48]. Furthermore, the morphology and crystal structure of the particles directly affect the exposure of active sites, the interaction with the substrate and, implicitly, the kinetics of the catalytic reaction [49]. Another important factor is the addition of heteroatoms or transition metals, which play an important role in controlling catalytic activity. The introduction of foreign elements into the crystal lattice can modify the electron density, charge distribution, and substrate affinity [50]. An example in this regard is the doping of manganese oxide with cobalt or copper has shown significant improvements in peroxidase activity and better stability in acidic media, suggesting a synergistic effect between the dopant atoms and the central metal atoms [63,64]. As expected, the structural defects and oxidation state can facilitate electron transfer and create additional reactive sites, essential aspects in simulated enzymatic catalysis processes [48].

Other factors that affect the activity of nanozymes are extrinsic factors, namely temperature, pH or the presence of other compounds in the medium. For example, the peroxidase activity of iron-based nanozymes is maximal in acidic medium, but decreases significantly under alkaline conditions [53]. Changes can be explained by protonation or deprotonation of functional groups on the surface of nanoparticles, which alter the affinity for the substrate and the reaction rate [54].

Temperature is another important factor, influencing molecular mobility and the frequency of collisions of the substrate with the catalytic surface, having the potential to accelerate catalytic reactions. However, too high temperatures can destabilize the structure of nanozymes, leading to aggregation or loss of functional activity [65,66].

Surface functionalization of nanozymes is a factor that influences their interaction with biological or industrial environments. Modification with polymers, organic ligands, or biomolecules can improve solubility in aqueous solutions, confer specificity for certain substrates, and protect against inactivation by aggregation or nonspecific adsorption. For example, coating with polyacrylic acid or polyethylene glycol has been shown to improve colloidal stability and reduce toxicity in biological applications [57].

The catalytic activity of nanozymes can also be modulated by the application of external physical factors, such as electric fields, light, or ultrasound. For example, piezoelectric nanozymes can generate surface charges under ultrasound, which directly participate in redox reactions, thereby enhancing catalytic activity. In other cases, irradiation with visible or UV light can activate photocatalytic nanozymes, such as those based on titanium dioxide or zinc oxide, which can produce active oxygen species useful in therapeutic or disinfection applications [44].

Therefore, the activity of nanozymes is the result of a complex interaction between their intrinsic properties (chemical composition, structure, morphology, and oxidation state) and external conditions (pH, temperature, surface functionalization, and physical stimuli).

### 3.3. Types of Nanozymes

**Figure 8:**
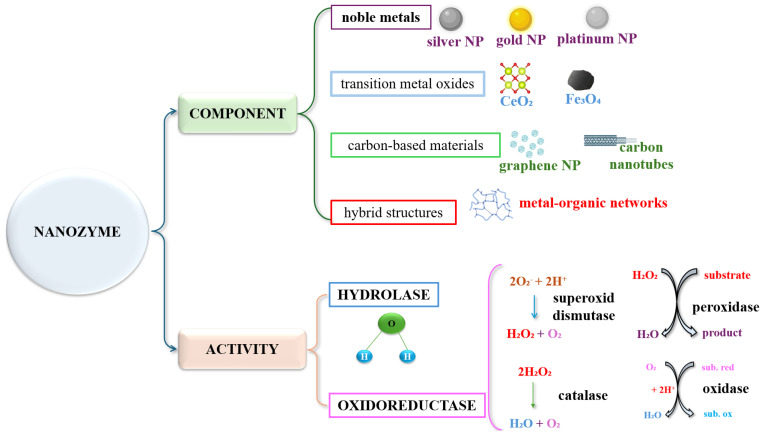
Types of nanozymes according to their component and activity; NP—nanoparticle, CeO_2_—cerium oxide, Fe_3_O_4_—iron oxide, O_2_—oxygen, H_2_O_2_—water, H/H^+—^hydrogen/proton, sub ox.—oxidized substrate.

When considering the types of nanozymes, it should be taken into account that they are divided into two broad categories depending on the active component and, consequently, depending on their catalytic activity [58] (Figure 8).

***Metal-based nanozymes*** are among the most studied. They include nanoparticles of noble metals (gold, silver, platinum) [67] as well as transition metals (iron, copper, manganese) [68]. Gold and platinum-based ones are known for their like-peroxidase and oxidase activity. Thus, AuNPs can catalyze oxidation reactions in the presence of hydrogen peroxide and are used in biosensors for the detection of glucose and other biomolecules [69]. The structure and size of these nanoparticles significantly affect the catalytic activity; reducing the particle size results in a larger specific surface area and consequently, higher catalytic efficiency. Structural engineering, which includes control of exposed crystal surfaces and doping with other metals, allows the adjustment of the enzymatic activity and specificity of these nanozymes [70].

In contrast, those based ***on transition metals***, such as iron and manganese, exhibit remarkable peroxidase and catalase activities [68]. Iron oxide nanoparticles can catalyze the decomposition of hydrogen peroxide, generating reactive oxygen species that are used in antitumor therapies and in anti-inflammatory drug therapies diseases [71].

Manganese oxides (MnO_2_) have also been shown to have peroxidase activity and are used in biomarker research and antioxidant therapy [64]. Metal oxides such as cerium oxide (CeO_2_) and MnO_2_ are used for superoxide dismutase and catalase activities. CeO_2_ NPscan mimic the activity of superoxide dismutase, scavenge reactive oxygen species, and protect cells from oxidative stress. These properties make them useful in biomedical applications, including the treatment of neurodegenerative diseases and antioxidant therapy [64].

***Carbon-based nanozymes*** include materials such as oxidized graphene, carbon quantum dots, and carbon nanotubes [72]. They exhibit multiple enzymatic activities, including peroxidase and superoxide dismutase, and are valued for their biocompatibility and stability. For example, carbon quantum dots have been used to protect cells against oxidative stress in anti-inflammatory therapies [73].

Carbon nanotubes and graphene exhibit peroxidase and catalase-like enzymatic activities and are used in the development of electrochemical biosensors and in antioxidant therapies. Their unique structure and large specific surface area allow for efficient interactions with biological substrates, and functionalization with specific chemical groups may enhance selectivity and catalytic efficiency [74].

Graphene oxide and graphene quantum dots have demonstrated remarkable enzymatic activities, including peroxidase and superoxide dismutase activity. These properties make them valuable in applications such as photodynamic therapy and reactive oxygen species detection, providing a promising alternative to natural enzymes under extreme pH and temperature conditions [75].

***Hybrid structures*** such as metal–organic framework-based nanozymes are porous materials composed of metal ions coordinated with organic ligands. Due to their high porosity and the possibility of functionalization, MOFs can mimic diverse enzymatic activities. For example, vanadium-based MOFs have proved peroxidase activity and have been used in biosensors for the detection of cancer biomarkers [76,77]. In therapy of cerebrovascular accident, MOF nanozymes have shown the ability to modulate reactive oxygen species (ROS) levels, reduce inflammation, and prevent neuronal apoptosis [78].

Nanozymes with ***hydrolase activity*** represent an emerging category of artificial catalysts capable of mimicking the hydrolysis reactions characteristic of essential natural enzymes, such as esterases, phosphatases, nucleases and proteases. Unlike their biological counterparts, these nanozymes exhibit exceptional physicochemical stability, can function in a wide range of temperatures and pH, and are distinguished by a higher reusability, qualities that recommend them for applications in biomedicine, industry and environmental remediation [79,80,81]. Structurally, hydrolase nanozymes are extremely diverse.

They can be synthesized in the form of inorganic nanomaterials (such as metal oxide nanoparticles), metal-organic frameworks (MOFs), and functionalized polymeric materials. Among these, zirconium-based MOFs have attracted particular interest, due to their ability to catalyze hydrolysis reactions similar to natural esterases, and their efficiency in the degradation of organophosphate compounds, including neurotoxic agents [80].

In the biomedical field, these nanozymes are often used to activate prodrugs in the tumor microenvironment, favoring the controlled release of active substances only in the affected tissues. This mechanism increases therapeutic efficacy and reduces systemic adverse effects, which is being explored in advanced oncological therapies [81]. In the field of environmental protection, hydrolase like nanozymes prove useful in the degradation of pesticides and resistant organic pollutants, as well as in the neutralization of toxic agents [79].

***Oxidoreductase nanozymes*** mimic the functions of natural enzymes involved in redox reactions, such as peroxidases, oxidases, catalases, and superoxide dismutases [62]. The structure of oxidoreductase nanozymes is varied, including metal nanoparticles, metal-organic frameworks, carbon-based materials, and hybrid composites [82]. In the biomedical field, oxidoreductase nanozymes have been used for biomarker detection, cancer therapy, and treatment of neurodegenerative diseases [11,83], and in environmental applications they have been explored for the degradation of organic pollutants and the detoxification of hazardous chemical agents [84].

Nanozymes offer a versatile and efficient platform for applications in biomedicine, environmental protection and beyond. Advances in the development and functionalization of these nanozymes are expanding their range of applications, highlighting their potential as sustainable and efficient alternatives to natural enzymes.

## 4. Iron Oxide Nanoparticles

### 4.1. Synthesis Methods

**Figure 9:**
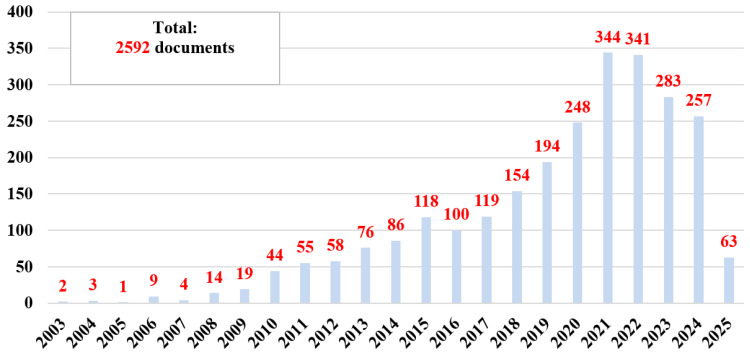
Number of publications related to the topic “iron oxide nanoparticles” in the Web of Science database.

Iron oxide nanoparticles (IONPs) have been intensively studied, especially in the last decade (Figure 9). The remarkable magnetic properties, biocompatibility, versatility and small size (10–20 nm) make iron oxide NPs ideal candidates for numerous applications [85]. These characteristics give them a central role in a variety of industrial, environmental, biomedical, therapeutic, diagnostic, biosensor, drug delivery, and biotechnological applications [86,87,88]. Iron oxides, especially magnetite (Fe_3_O_4_) are abundant inorganic materials and are the most intensively studied in terms of size, morphology, oxidation state and crystal structure [89].

**Figure 10:**
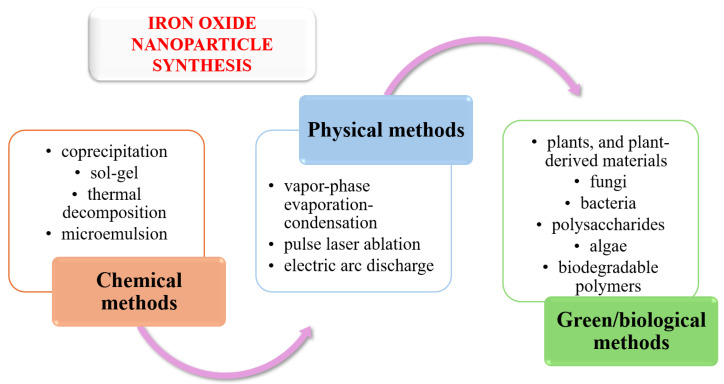
Main methods of iron oxide nanoparticles synthesis.

Although various classifications can be found in the specialized literature, the methods of iron oxide nanoparticle synthesis follow three main directions: chemical, physical and biological [83,84,85] (Figure 10). Chemical methods (co-precipitation, sol-gel, thermal decomposition and microemulsion methods) are the most widely used, as they allow a precise a priori control of particle size and morphology [86]. Green or biological methods use plant extracts or microorganisms to reduce iron salts, which is an environmentally friendly and biocompatible alternative [78,85]. Physical methods for the synthesis of iron oxide nanoparticles include physical processes that produce nanoscale particles without direct chemical intervention. These methods are recognized for their ability to produce nanoparticles of high purity and controlled size and morphological distribution. The most commonly used methods are vapor-phase evaporation–condensation, pulsed laser ablation, and electric arc discharge [87,88].

Iron oxide nanoparticles represent an important class of nanomaterials with numerous applications in biomedicine, catalysis, environment and electronics due to their unique magnetic, chemical and structural properties. To obtain nanoparticles with well-controlled size, morphology and functional properties, several synthesis methods have been developed, tested and analyzed, each with specific advantages and limitations. The co-precipitation method, for example, is one of the most widely used techniques due to its simplicity, low cost and high yield, but the poor control of particle size and the tendency to form aggregates represent significant drawbacks [88]. In contrast, the thermal decomposition method offers excellent control over nanoparticle size and shape, resulting in monodisperse particles with superior crystallinity. However, it requires high temperatures and the use of toxic organic solvents, which limits its applicability in biological environments [83].

Physical methods such as laser ablation have the advantage of obtaining pure particles free of chemical contamination, but require expensive equipment and produce low yields, making their application on an industrial scale difficult [88]. In contrast, green synthesis, which uses plant extracts or microorganisms to reduce metal ions, is gaining importance. This method is environmentally friendly and yields biocompatible particles, but the control of size and shape is limited and the reproducibility of the process is often poor [78,85,89]. Table 2 shows some key features of several techniques used in the synthesis of iron oxide nanoparticles.

### 4.2. Characterization Methods of Iron Oxide Nanoparticles

The characterization of iron oxide nanoparticles is essential in understanding their physical, chemical, structural and functional properties, which are crucial to validate applications in fields such as biomedicine, catalysis, industrial and molecular biotechnology. A wide range of characterization techniques are used to obtain a complete evaluation of these materials, each providing complementary information on size, morphology, crystal structure, chemical composition, magnetic properties and colloidal stability [83].

Transmission electron microscopy (TEM) and scanning electron microscopy (SEM) are standard methods for determining the morphology and size of nanoparticles. TEM allows visualization at the atomic level and provides detailed information on particle shape and size distribution, while SEM is used to analyze the topography and agglomeration of nanoparticles on the surface [83].

X-ray diffraction (XRD), meanwhile, is used to determine crystal structure of nanomaterials, providing essential information about the atomic order and crystallite size. In the case of iron oxide nanoparticles, XRD allows the evaluation of the degree of crystallinity and the estimation of crystal sizes, thus constituting an indispensable tool in structural characterization. Although phases such as magnetite and maghemite have very similar structures and lattice parameters, which can complicate the interpretation of the data, XRD remains the main method for the structural analysis of these nanoparticles, and the results obtained can be correlated with other spectroscopic methods for a complete characterization [91,92]. Fourier transform infrared (FTIR) spectroscopy is used to detect functional groups on the surface of nanoparticles and to assess interactions with functionalizing agents, polymers or bioconjugates. This technique is essential for characterizing nanoparticles intended for biomedical applications [93]. Another useful characterization method is dynamic light scattering (DLS). It is essential for determining the hydrodynamic size of nanoparticles in suspension, providing information on size distribution and colloidal stability [94]. Table 3 describes the main techniques used to characterize iron oxide nanoparticles.

### 4.3. Sources of Iron Oxide Nanoparticles

The use of iron oxide nanoparticles depends directly on their properties and quality. Properties such as crystal size and uniformity are important and decisive aspects that are closely linked to specific applications. In addition, biocompatibility and magnetic properties are other aspects that determine the direction of use of these particles.

In this respect, the type of particles obtained by different synthesis routes has a different impact on the orientation of applicability. At the same time, in most cases, the type of IONP synthesis is closely linked to the source of these particles [96].

A common source of IONPs is the co-precipitation method. This involves the reaction of iron salts (Fe^2+^ and Fe^3+^) in an alkaline medium to form magnetite particles (Fe_3_O_4_) [97]. Another method involves the decomposition of organometallic iron precursors in organic solvents, often in the presence of surfactants, at elevated temperatures. This method produces nanoparticles of well-controlled size and shape, such as spheres and prisms [88].

Another source is the continuous flow synthesis of these IONPs. The latter has gained attention due to the possibility of scaling and precise control of the size of the nanoparticles. A notable example is the use of a reactor with an adjustable thermal profile, allowing the synthesis of IONPs with sizes between 2 and 17 nm, suitable for biomedical applications such as magnetic resonance imaging and magnetic hyperthermia [99].

A distinct source is the production of IONPs through green synthesis. These processes use plant extracts or microorganisms to reduce iron ions and form nanoparticles. This approach is environmentally friendly and produces nanoparticles with increased biocompatibility. An example of this is the production of iron oxide nanoparticles from the cyanobacteria *Leptolyngbya foveolarum* and the bacterium *Azospirillum brasilense*. The particles obtained in this way have proven useful in wastewater treatment [100].

Another example is the synthesis of IONPs using plant extracts, which showed antioxidant activity and cytotoxicity against cancer cells, highlighting the potential of these nanoparticles in biomedical applications [101].

An alternative source of IONPs, explored in the context of the circular economy, in order to increase sustainability and protect raw materials, refers to the synthesis of magnetic iron oxide nanoparticles from the recycling of steel waste [100]. Thus, Table 4 summarizes the main sources of IONPs together with the most important limitations of their use.

### 4.4. Magnetotactic Bacteria—Source of Naturally Synthesized Iron Oxide

#### 4.4.1. Magnetosome Formation and Magnetic Crystals Biomineralization in MTB

**Figure 11:**
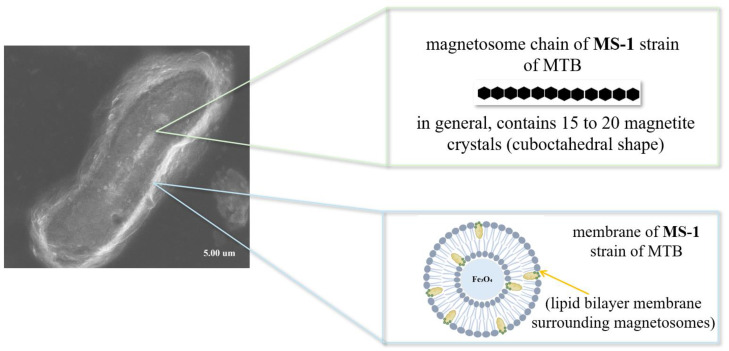
Scanning electron microscopy image of MTB (*MS-1* strain) (**left**), magnetosome membrane and magnetosome chain strain (**right**); MTB—magnetotactic bacteria.

MTB are a special group of prokaryotic organisms. They have the ability to move and orient themselves according to the Earth’s magnetic field thanks to the presence of their intracellular structures [104,105,106]. These intracellular structures are actually small magnetic crystals biomineralized in the MTB membrane, called magnetosomes [104,105]. Magnetosomes (Figure 11) are membrane-bound organelles containing magnetite (Fe_3_O_4_) or greigite (Fe_3_S_4_) crystals, organized into chains, which give MTBs a stable magnetic moment and allow them to navigate efficiently to areas with optimal oxygen concentrations [101]. The process of magnetosome biomineralization is a remarkable example of biological control over the formation of nanostructures. It involves the invagination of the inner membrane to form precursor vesicles, followed by the accumulation and conversion of iron into magnetite or greigite crystals [104].

In recent decades, research on MTBs has highlighted their potential in a variety of fields, from biomedicine to environmental remediation. Magnetosomes produced by these bacteria present significant advantages over synthetically produced magnetic nanoparticles due to their uniformity, biocompatibility, natural functionalization with biological membranes, and magnetic stability [18,105,106]. Their applications in biomedicine include targeted drug delivery, magnetic resonance imaging (MRI), magnetic hyperthermia for cancer treatment, cell separation, and recently their role in the development of biosensors has been highlighted [101,106,107,108].

**Figure 12:**
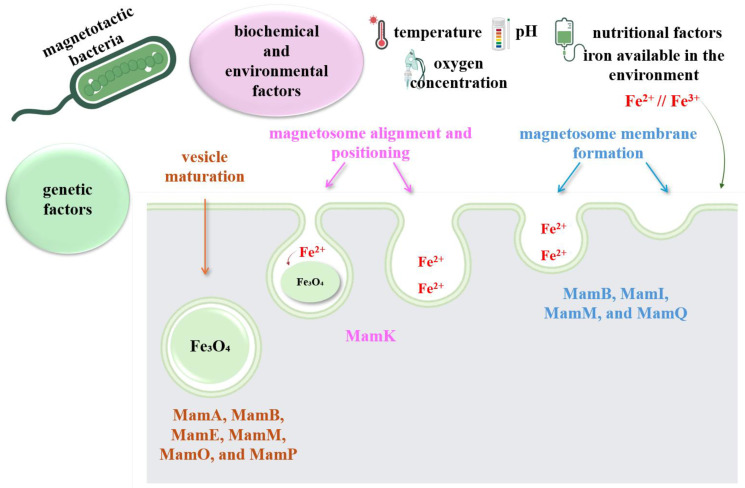
Genetic, biochemical and environmental factors that influence the magnetosome formation and their mineralization; MamA, MamB, MamE, MamM, MamO, MamP, MamK, MamQ—magnetosomal proteins involved in magnetsome formation, alignment and vesicle formation.

Although the process of magnetic crystal mineralization, or magnetosome formation, appears to be straightforward, it is sophisticated and is regulated by genetic, biochemical and environmental factors (Figure 12) [102]. Genetic control is exerted through the action of translational products of genes associated with magnetosome membrane formation (MamB, MamI, MamM and MamQ), genes involved in magnetosome alignment and positioning (MamK) and genes involved in vesicle maturation (MamA, MamB, MamE, MamM, MamO and MamP) [109].

Regarding biochemical and environmental factors, they are closely linked to nutritional factors, the amount of iron available in the environment, temperature, pH and oxygen concentration. These factors influence both the adaptation of bacteria to environmental conditions and the mineralization process, either in terms of MTB development or in terms of inhibiting the mineralization process and, implicitly, their development [107,108].

The mineralization of magnetic crystals ensures that MTB acquires directional magnetic properties, while also providing a stable magnetic moment [107]. In this way, MTB possesses the ability to actively orient itself, depending on the terrestrial magnetic field (magnetotaxis) [109], in water columns or sediments to identify areas with optimal oxygen (low concentrations, between 0.1–0.5 mg/L) [109] and redox conditions [110].

On the other hand, mineralization in magnetosomes brings a structural and functional advantage to MTB. The process is strictly controlled at the molecular level, which leads to the formation of uniform crystals in terms of size, shape and crystallographic orientation, critical elements for the magnetic stability of the particles.

These characteristics ensure that magnetosomes function efficiently as an intracellular compass, without energy loss through magnetic fluctuations [109,111]. In addition to the direct biological function, mineralization is responsible for the generation of extremely pure and morphologically well-defined nanoparticles, making them extremely valuable for biotechnological and biomedical applications [22]. Unlike synthetic nanoparticles, those biomineralized in MTB exhibit superior biocompatibility, functionalizable surfaces and stability in biological environments, due to their lipid shell derived from the bacterial membrane. In addition, it also enables controlled doping of crystals with other elements (e.g., cobalt, manganese, gadolinium) [112], which deliberately changes their magnetic or optical properties, depending on the intended applications (e.g., drug delivery, magnetic hyperthermia) [34,113,114].

Magnetosome production by magnetotactic bacteria is an area of growing interest due to their applications in nanotechnology and biomedicine. In general, the yields obtained depend on the culture conditions, the type of medium used and the optimization of growth parameters. The production throughput can be increased by using controlled fermenters and optimized media, but the associated costs remain an important limitation, influenced by the ingredients of the medium and the duration of cultivation. Scalability of the process is possible, but encounters bottlenecks such as the complex metabolism of the bacteria and the difficulties in separating and purifying magnetosomes without compromising the integrity of the particles. Despite these challenges, magnetosome production continues to be a promising target for industrial and biomedical applications, requiring special attention to process optimization to increase yields and reduce costs [115,116,117,118].

In the research on magnetosome production by magnetotactic bacteria, various strains were investigated to evaluate yields and optimal culture conditions. For instance, the strains *Magnetospirillum gryphiswaldense* MSR-1, *Magnetospirillum magneticum* AMB-1, *Magnetospirillum* sp. ME-1 and *Magnetospirillum magnetotacticum* MS-1 were studied in batch and fed-batch cultures, with the aim of determining the optimal parameters for magnetosome production [115,119]. For example, Ke et al. [119] controlled the optical density (OD), to enhance the production of magnetosomes, at a constant level of 0.5% by coupling to the air-flow rate and stirring rate. Thus, using the strain *Magnetospirillum* sp. ME-1, they obtained a cell density and magnetosome yield at 49 h were 6.5 (OD565) and 120 mg L^−1^ (wet weight) [119]. In the case of MSR-1, another group of researchers, Liu et al. [116], obtained an OD of 12.3 using the fed-batch method, but with a cultivation period of 36 h [116]. Another example is the study by Yang et al. [120], who cultivated AMB-1 cells in magnetic spirillum growth medium enriched with L-cysteine, yeast extract, and polypeptone. L-cysteine enhanced cell growth and reduced lag phase, resulting in high magnetosome production with a final cell density of 0.34 g cell dry weight/l [120].

A comparative study conducted by Heyen and Schüler [121] on three strains of Magnetospirillum (*M. gryphiswaldense*, *M. magnetotacticum* and *Magnetospirillum* sp. AMB-1) aimed at determination of optimum growth conditions and the establishment of a reliable method for mass cultivation in flasks and the fermentor and reported the existence of some differences in the response to oxygen concentrations. However there is one common point, namely that the formation of magnetosomes was triggered only below a threshold level of dissolved oxygen (pO_2_ < 20 mbar), and the optimal condition for magnetite biomineralization was at a pO_2_ of approximately 0.25 mbar. The highest magnetosome productivity (6.3 mg magnetite per liter per day) was obtained with *M. gryphiswaldense* in a fermentor with automated oxygen control (oxystat), representing the highest yield reported at that time for a magnetotactic bacterium, thus providing a solid basis for large-scale cultivation under definable conditions [121].

The production of magnetosome-derived iron oxide nanoparticles still involves significant costs, even though their structural and functional benefits, such as nanoscale uniformity, biocompatibility and high functionalization potential, justify the intense interest in this technology. Techno-economic analyses indicate an estimated production cost of 10,372 USD/kg in a single-step fed-batch process and 11,169 USD/kg for semi-continuous processes, and the resulting minimum selling price ranges between 21 and 120 USD/g, depending on the technological parameters and production scale. These costs are almost two orders of magnitude lower than those of magnetite nanoparticles, even highly functionalized, which trade in the range of 10,000–11,000 USD/kg, a significant difference compared to traditional chemical synthesis [118]. However, optimization of culture media shows a real potential to reduce the costs associated with magnetosome production; the yield of magnetosomes can increase up to eightfold, while maintaining the economic efficiency of the process [117]. Thus, although biological production remains more expensive compared to chemical methods, the differences can be reduced through optimization, and the added value conferred by the intrinsic qualities of magnetosomes keeps them competitive for advanced applications in medicine and nanotechnology.

In this regard, kinetic parameters (such as Km, Vmax and kcat) provide essential information about the substrate affinity and catalytic efficiency of nanoparticles, being critical factors in assessing their potential for biomedical and biosensing applications. The peroxidase-like activity of iron oxide nanoparticles requires certain (optimal) conditions, similar to those for HRP. These conditions refer to temperature (optimal at 37–40 °C), pH (optimal pH 3–6.5) in an acidic buffer solution (the most commonly used being 3,3′,5,5′-Tetramethylbenzidine-TMB or 2,2′-Azino-bis(3-ethylbenzothiazoline-6-sulfonic acid)-ABTS) [122,123,124]. In this context, the differences between synthetic nanoparticles and those biomineralized by magnetotactic bacteria become more evident when analyzed in terms of these parameters, as seen in the table below (Table 5).

#### 4.4.2. Advantages of Magnetite Crystals from MTB

**Figure 13:**
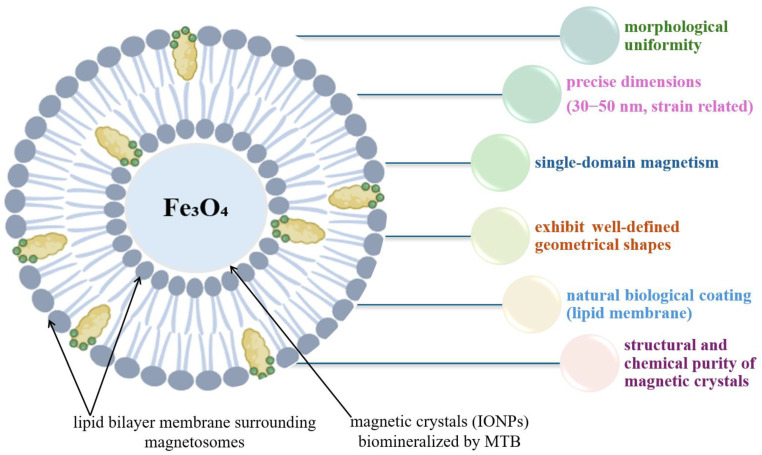
High-quality biomineralized nanoparticles made by magnetic bacteria; MTB—magnetotactic bacteria, IONPs—iron oxide nanoparticles (Fe_3_O_4_).

Magnetotactic bacteria represent a fascinating class of microorganisms capable of synthesizing, in a controlled manner, magnetite nanoparticles within their cells. These crystals are a model of excellence in the natural synthesis of nanomaterials. In a context dominated by the excessive demand for high-performance magnetic nanoparticles in numerous fields, from biomedicine to nanotechnology, MTB biosynthesized magnetite stands out for a number of essential advantages over its synthetic analogues obtained chemically or physically, Figure 13.

One of the most significant advantages is the morphological uniformity of the nanoparticles. The magnetite produced intracellularly by MTB has remarkably precisely controlled sizes, typically between 30 and 50 nm, corresponding to the single-domain magnetism regime, a necessary condition for achieving stable magnetization. These sizes are the result of rigorous genetic and biochemical control exerted by bacteria on the process of crystal nucleation and growth. Unlike chemical synthesis methods, where the size distribution is often broad, biosynthesized magnetite exhibits a high monodispersity, which ensures consistency and reproducibility in technological applications [116,117]

The crystal shape is another important aspect. Magnetosomes show well-defined geometries (cubic, prismatic, ovoid) dictated by structural proteins such as Mms6 or MamC. These shapes not only influence the intrinsic magnetic properties of the nanoparticles, but also contribute to coherent alignment in intracellular chains, maximizing the net magnetic moment of the cell. The chain-like organization reduces the disordered dipole interactions that often occur in synthetic nanoparticle aggregates, thus favoring predictable and stable magnetic behavior [102,104].

In addition to its exceptional physicochemical properties, biosynthesized magnetite has a natural biological coating in the form of a lipid membrane. This membrane provides enhanced biocompatibility and colloidal stability, two essential features in biomedical applications such as targeted drug delivery, magnetic resonance imaging (MRI), or magnetic hyperthermia treatments [18,116,118,119]. In comparison, synthetic nanoparticles require additional functionalization to achieve the same level of biological integration.

Another notable advantage of magnetite produced by MTB is its structural and chemical purity [116]. Bacteria use natural mechanisms to exclude impurities and maintain a precise crystalline composition, whereas chemical methods are susceptible to contamination or the formation of secondary phases. Therefore, bacterial magnetite is ideal for applications requiring uniform and finely controlled magnetic properties. The biological production of these crystals offers ecological and sustainable advantages, as the process occurs under mild temperature and pH conditions, without the need for toxic reagents or high energy consumption. Genetic control of biosynthesis also opens up promising prospects in metabolic engineering and in the scalable production of magnetosomes through heterologous expression in model microorganisms.

## 5. Why Use Iron Oxide Nanoparticle-Based Biosensors Instead of Conventional Enzymatic Biosensors?

In the context of the development of high-performance biosensors for biomedical, food and environmental applications, a conceptual and technological differentiation is noted between traditional enzyme-based platforms and emerging ones using nanomaterials, particularly iron oxide nanoparticles.

Enzymatic biosensors, developed for the detection of analytes such as glucose, lactate, or hydrogen peroxide, are based on biochemical reactions catalyzed by enzymes immobilized on the surface of an electrode. The advantages of these systems include high specificity, operation under mild conditions, and easy integration into electrochemical or optical platforms. However, these devices have significant limitations related to enzyme stability, sensitivity to environmental conditions (pH, temperature), and biodegradation over time, affecting the durability and reproducibility of the sensors [120,121,122].

In contrast, the use of iron oxide nanoparticles (especially magnetite) opens up novel perspectives in the field of biosensing and offers alternative or complementary solutions to enzymatic platforms. These nanomaterials possess unique physicochemical properties (such as superparamagnetism, good conductivity, biocompatibility, and ease of functionalization) that give them a great potential for integration into multi-modal detection devices. Unlike enzymatic biosensors, iron oxide-based systems enable the pre-concentration of analytes by applying external magnetic fields and favor signal amplification by increasing the active area or by facilitating the transport of electrical charges [126].

Compared to enzymatic biosensors (in which the signal is strictly dependent on the catalytic activity of the biocatalyst), iron oxide nanoparticle biosensors can integrate more complex detection mechanisms, including direct electrochemical processes, molecular recognition interactions through aptamers, or coupling reactions with optical or fluorescent indicators [127]. This versatility results in a substantial increase in sensitivity, especially in the detection of very low concentration biomarkers, such as microRNAs, tumor antigens, or cardiac proteins. Another distinctive aspect is the hardness and stability of iron oxide-based systems. Although enzymatic biosensors are often limited to short-term uses, mainly due to the loss of enzymatic activity through denaturation or inhibition, magnetic biosensors have higher resistance to temperature, pH, or biological contamination.

Furthermore, chemical functionalization of nanomagnetic surfaces with stable recognition molecules allows the preservation of sensor activity over extended periods, which is essential for applications in complex environments or in portable diagnostic devices [128,129,130].

An overview of the main advantages and limitations of both types of biosensors is summarized in Table 6.

Iron oxide nanoparticle-based biosensors offer a robust and scalable alternative to traditional enzymatic biosensors, highlighting their stability, versatility and potential for miniaturization. Although enzymatic biosensors remain relevant in applications where catalytic specificity is essential, emerging technologies based on magnetic nanomaterials offer significant advantages in terms of sensitivity, durability and integration into advanced diagnostic devices [146]. The evolution of these platforms suggests a trend of convergence between the two paradigms, with an emphasis on the development of hybrid biosensors that combine the biochemical specificity of enzymes with the structural and functional performance of nanomaterials.

## 6. Iron Oxide-Based Nanozymes in a New Generation of Biosensors and Their Unique and Improved Catalytic Properties in the Presence of Various Added Nanomaterials

### 6.1. Using Iron Oxide NPs

Iron-based nanoparticles (notably magnetite Fe_3_O_4_ and hematite Fe_2_O_3_) have emerged in recent years as key nanomaterials in the development of next-generation biosensors, due to their remarkable physicochemical properties and functional versatility. Characterized by superparamagnetic behavior and a functionalizable surface, these particles facilitate the efficient integration of biological recognition elements into the sensor architecture, allowing the specific and ultrasensitive detection of various biomolecules of interest, particularly in the medical, food or environmental fields [138,147].

Advances in synthesis technology have led to the production of iron nanoparticles with controlled size, uniform morphology and high dispersibility, properties essential for the reproducibility and performance of biosensors. Synthesis techniques (such as coprecipitation or sol-gel technique) allow precise modification of surface properties, in order to selectively attach antibodies, aptamers, nucleic acids or other recognition molecules. Functionalization of nanoparticles with specific ligands or polymeric coatings determines increased stability in complex biological solutions and ensures the biochemical compatibility required especially in clinical applications [90,128,148].

The broad applicability of iron nanoparticles is evidenced by the successful detection of microRNAs, cancer-associated antigens, toxic metal ions or enzymes involved in acute myocardial infarction using biosensors that combine electrochemical and magnetic properties. Furthermore, the performance of these systems can be improved by combining them with hybrid nanocomposite structures (graphene or gold nanoparticles) that further contribute to the efficiency of electron transfer and the increase in the loading capacity of the bioreceptors [128,129,130,149].

Although the potential of these nanomaterials is considerable, challenges related to biocompatibility, colloidal stability, and operation in real environments persist. It is necessary to optimize the engineering of crystal defects, which influence magnetic and electrochemical behavior, as well as implement robust methods of anchoring bioreceptors to reduce nonspecific interactions and functional degradation. In parallel, efforts at miniaturization and integration into wearable devices are essential for the transition of iron nanoparticle-based biosensors to widespread clinical applications [129,130,150,151].

### 6.2. Using Hybrid Iron Oxide-Gold NPs

**Figure 14:**
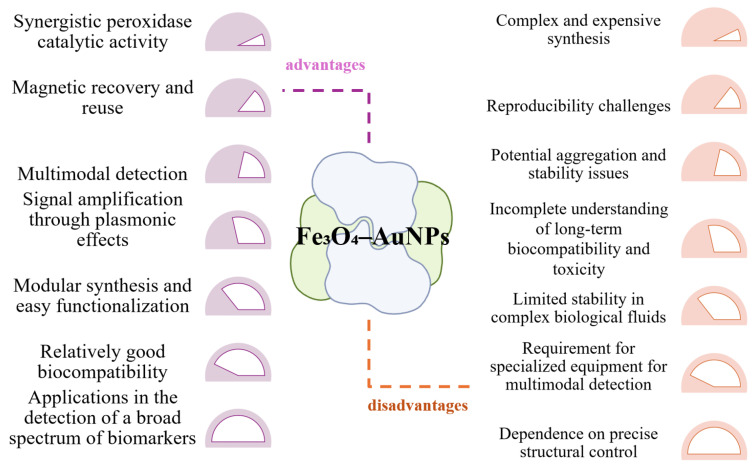
Advantages and disadvantages of hybrid nanostructures of magnetite and gold nanoparticles in the construction of biosensors; Fe_3_O_4_-AuNPs—iron oxide and gold nanoparticles.

Nanozymes consisting of hybrid iron oxide–gold nanoparticles represent an emerging class of artificial enzymes that combine the magnetic and redox properties of iron oxide with the plasmonic and catalytic functionalities of gold. These bimetallic structures are particularly valuable in the field of biosensors due to their enhanced peroxidase activity, which allows them to equal or even surpass the efficiency of natural enzymes [152]. In this combination (Figure 14), magnetite provides peroxidase-like activity, facilitating the decomposition of hydrogen peroxide under mild conditions, while gold nanoparticles contribute to signal amplification through plasmonic effects and to the anchoring of specific biomolecules. The resulting hybrid structures exhibit a synergistic effect, in which the total catalytic activity exceeds the sum of the individual contributions of the two components [153,154].

The integration of the two materials into a single nanozyme entity allows for highly efficient catalytic platforms that can be used in a variety of sensing formats, including colorimetric, electrochemical, or surface enhanced RAMAN spectroscopy (SERS) detection [155]. The presence of magnetite allows for magnetic manipulation and separation of the biosensor, which facilitates its recovery and reuse, which is essential for the development of sustainable and low-cost systems. The ability of hybrid nanozymes to generate intense optical signals, due to gold surface plasmons, offers a major advantage in increasing sensitivity and lowering detection limits in assisted colorimetric analyses, including in portable devices connected to smartphones. However, there are also challenges associated with these materials [156]. The controlled synthesis of hybrid Fe_3_O_4_-Au nanoparticles requires complex methods, often expensive and difficult to scale up industrially, and obtaining a uniform distribution of gold on the magnetite surface is crucial for catalytic reproducibility. Also, despite the apparent biocompatibility advantages, prolonged exposure to iron and gold nanoparticles may generate oxidative stress or unpredictable effects in biological systems, which is why additional in vivo toxicity studies and assessments of the impact on human health and the environment are needed [155,157].

### 6.3. Using Hybrid Iron Oxide-Silver NPs

**Figure 15:**
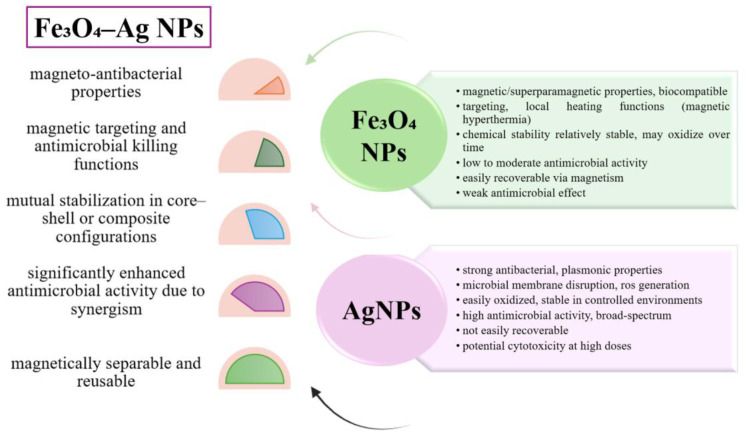
Key properties of hybrid nanostructures of magnetite and silver nanoparticles in the construction of biosensors; Fe_3_O_4_-AgNPs—iron oxide and silver nanoparticles, Fe_3_O_4_—iron oxide nanoparticles, AgNPs—silver nanoparticles.

Another example is the use of hybrid iron oxide (especially Fe_3_O_4_) and silver nanoparticles (AgNPs) (Figure 15). This combination has attracted increasing attention in recent years due to its synergic effect in various applications, from decontamination to antimicrobial therapies [158,159]. Iron oxide nanoparticles have attracted a lot of attention, being appreciated for their magnetic properties, the ability to induce magnetic hyperthermia, and the ease of immobilization on support loads (e.g., in hydrogels or aerogel materials) [160,161]. In contrast, AgNPs are known for their potent bactericidal effect, generating reactive oxygen species and releasing Ag^+^ ions that disrupt the cell membrane and microbial metabolism [162,163].

The association of these two classes of nanoparticles allows the obtaining of hybrid structures such as Ag-Fe_3_O_4_ core–shell or Ag-Fe_3_O_4_ composites in hydrogel, which capitalize on the advantages of both components. A relatively recent example is the use of Ag-Fe_3_O_4_ core–shell, produced by chemical reduction and co-precipitation processes. This use demonstrated significantly lower minimum inhibitory concentrations (MIC) against *S. typhimurium* and *Escherichia coli* compared to AgNP or Fe_3_O_4_NP used separately [164].

Moreover, magnetic hydrogels impregnated with AgNPs showed a dual antibacterial effect amplified by magnetic hyperthermia in the presence of an oscillating field [165], and in the context of wastewater treatment, green Ag–Fe–ZnO composites (obtained with plant extract) exploited the electron-donating capacity of FeNPs and the photo-oxidative effect of Ag and ZnO ions for the degradation of pharmaceutical contaminants [166].

Another promising approach is the fabrication and use of bifunctional magnetic-plasmonic nanoparticles, including Ag-Fe_3_O_4_, which offer magnetic control and optical (plasmonic) response, usable in biosensors, magneto-optical separation, SERS diagnostics, and photothermal therapies or magnetic hyperthermia [167,168].

### 6.4. Using Hybrid Iron Oxide-Copper NPs

**Figure 16:**
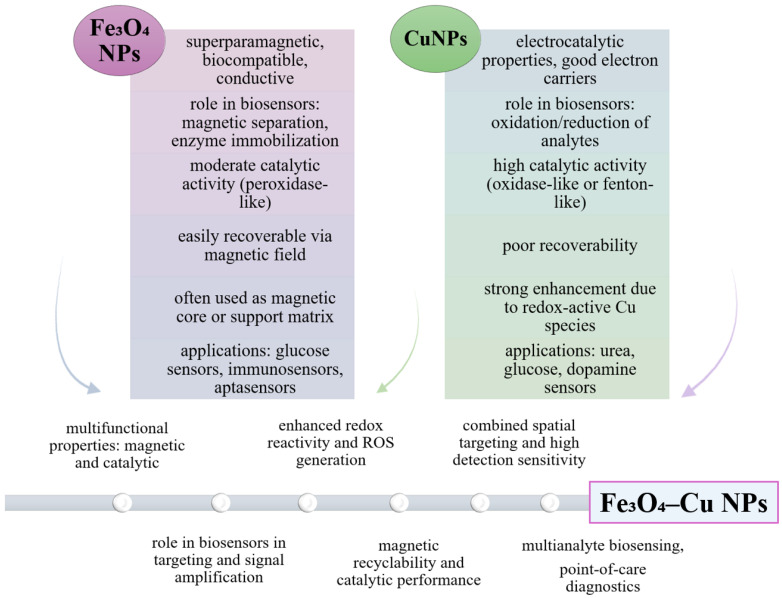
Key properties of hybrid nanostructures of magnetite and copper nanoparticles in the construction of biosensors; Fe_3_O_4_—iron oxide nanoparticles, CuNPs—copper nanoparticles.

The use of hybrid iron oxide–copper nanoparticles in biosensor design brings valuable synergies between magnetic and catalytic properties (Figure 16). Iron oxide nanoparticles are valued in biosensor construction for their superparamagnetism, their ability to facilitate separation and retention of the enzyme or biomarker on the sensor surface, and their peroxidase-like catalysis effect, useful in electrochemical signal amplification. In contrast, copper compounds or copper oxides (CuO/Cu_2_O) are recognized for their superior electrocatalytic activity in oxidation reactions (e.g., glucose, urea), as well as for their rapidly reversible Cu(II)/Cu(I) redox reactions, which improve the electrochemical signal and sensor sensitivity [169,170,171].

Copper-doped iron oxide nanoparticles have catalytic properties, generating reactive oxygen species that can amplify the electrochemical or optogenetic response in the presence of target biomarkers, especially at elevated temperatures. This aspect is suggestive of utility in dual catalysis-redox biosensors [172,173]. The role of complementation is embodied in the fact that Fe_3_O_4_ compensates for the sensor’s recoverability and stability, allowing magnetic separation and easy reuse of the electrode, while copper brings superior sensitivity and specificity for target molecules (e.g., glucose or urea). In addition, Cu doping in the iron oxide structure confers a Fenton-like catalytic activity, enhancing the chemical transformation of the analyte and amplifying the sensory signal [172,173].

### 6.5. Using Hybrid Iron Oxide-Platinum NPs

**Figure 17:**
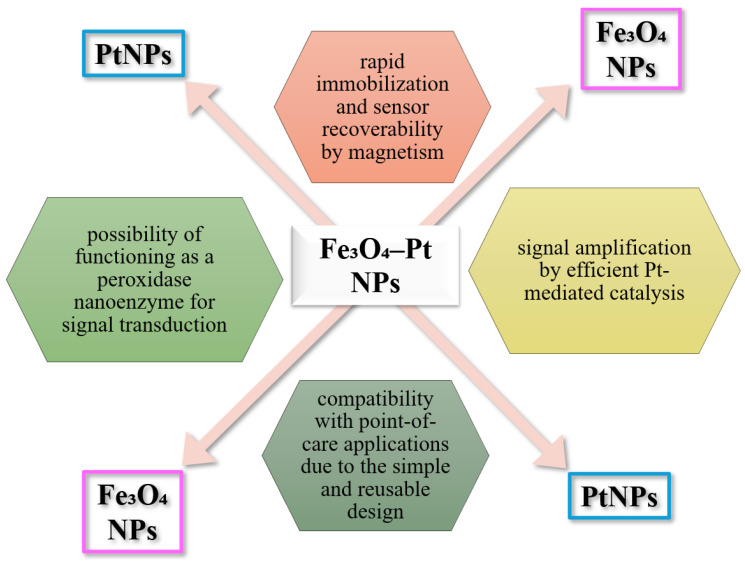
Key properties of hybrid nanostructures of magnetite and platinum nanoparticles in the construction of biosensors; Fe_3_O_4_—iron oxide nanoparticles, PtNps—platinum nanoparticles, Fe_3_O_4_ –PtNPs iron oxide and platinum nanoparticles.

Hybrid Fe_3_O_4_-Pt NP systems, especially core–shell ones, have rapidly evolved in recent years in electrochemical and optical biosensing due to the synergy between the magnetic properties of Fe_3_O_4_ and the catalytic characteristics of platinum particles (Figure 17). Fe_3_O_4_ provides magnetic support for rapid and selective immobilization of the analyte or enzyme, by applying an external magnetic field, and facilitates recurrent electrode recovery, being an essential advantage for testing in complex environments. In addition, Fe_3_O_4_ acts as a nanoenzyme with peroxidase-like activity, contributing to the generation of electrochemical signal in the presence of hydrogen peroxide (H_2_O_2_).

Deposition of a thin layer of Pt on the surface of Fe_3_O_4_ enhances the catalytic activity, in particular in redox and hydrogen peroxide oxidation reactions. Such Fe_3_O_4_-Pt structures have been successfully used in the rapid detection of glucose by colorimetric methods (e.g., in serum and urine) [174,175].

The defining features of the hybrid use of Fe_3_O_4_ and Pt include rapid immobilization and sensor recoverability by magnetism, signal amplification by efficient Pt-mediated catalysis, the possibility of functioning as a peroxidase nanoenzyme for signal transduction, and compatibility with point-of-care applications due to the simple and reusable design. These systems are suitable for the detection of glucose, H_2_O_2_, nitrite, or other redox-sensitive analytes in biological or environmental media [174,175,176].

### 6.6. Using Hybrid Iron Oxide-Carbonaceous NPs

**Figure 18:**
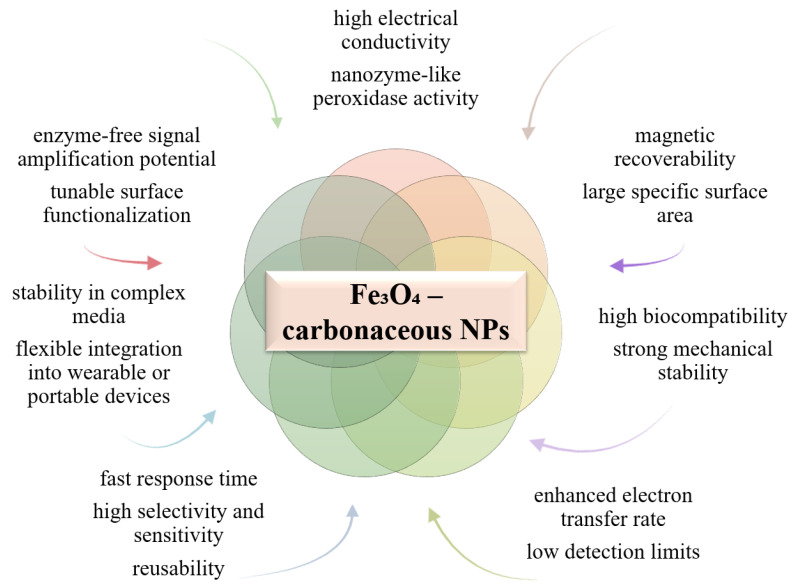
Hybrid nanostructures of magnetite and carbonaceous nanoparticles in the construction of biosensors; Fe_3_O_4_—iron oxide nanoparticles.

Use of hybrid iron oxide–carbon-based nanoparticles (such as graphene or carbon nanotubes) offers efficient hybrid qualities and properties in the design of electrochemical and optical biosensors through the synergy between magnetic and conductive properties (Figure 18). Fe_3_O_4_ ensures efficient immobilization and rapid recovery of the bioactive component by magnetization, as well as peroxidase activity that amplifies the detection signal. Carbon-based materials contribute with a large specific surface area, excellent conductivity and electrochemical stability, facilitating the transfer of electrons between biomolecules (enzymes, aptamers) and the electrode, which leads to increased sensitivity and response speed [177,178].

Some such examples are Fe_3_O_4_/graphene/chitosan hybrid biosensors for the detection of lead (Pb^2+^) in water [179], Fe_3_O_4_-C (amorphous carbon) with peroxidase nanozyme activity comparable to HRP (horseradish peroxidase), used in colorimetric glucose detection [156], and flexible Fe_3_O_4_/graphene sensors for the detection of H_2_O_2_ [156]. Thus, the Fe_3_O_4_–carbon hybrid structure provides a robust, easily recoverable and highly sensitive option, ideal for the detection of metal ions, biological analytes and neurotransmitters in complex environments, with potential for point-of-care applications [177,178,180].

### 6.7. Using Hybrid Iron Oxide-Indium Tin Oxide NPs

**Figure 19:**
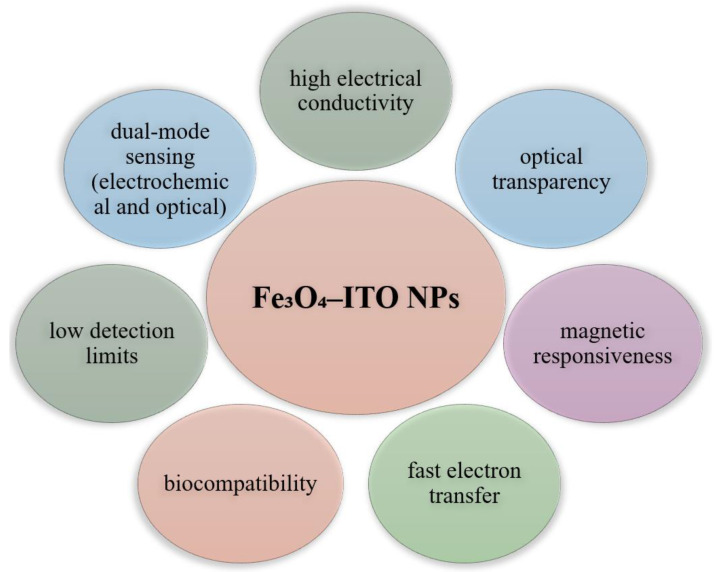
Hybrid nanostructures of magnetite and indium tin oxide nanoparticles for the construction of biosensors; Fe_3_O_4_—iron oxide nanoparticles, ITO NPS—indium tin oxide nanoparticles.

Iron oxide nanoparticles combined with indium tin oxide (ITO) layers on transparent electrodes constitute an innovative strategy in the development of electrochemical and optoelectronic sensors (Figure 19). Fe_3_O_4_ serves as a magnetic support for the rapid and selective immobilization of biomarkers or enzyme on the ITO surface, facilitating the recovery and reuse of the sensing platform, as well as signal amplification due to its peroxidase activity. At the same time, the ITO layer brings a series of essential benefits: excellent electrical conductivity, optical transparency that allows the integration of photometric techniques and flexibility in the design of lab-on-chip sensors or implantable devices [138,181].

The Fe_3_O_4_–ITO hybrid structure maximizes the synergy between the two materials: Fe_3_O_4_ provides the magnetic trapping mode and electrochemical transduction via redox reactions, while ITO provides optimal conductive and optical support, enabling the measurement of both electrical current and optical signals in integrated biosensors [182]. This combination holds promise for the development of fast, sensitive, and reusable sensing platforms in a variety of biomedical, environmental, and point-of-care diagnostic applications.

## 7. Discussions and Future Research Directions

Iron oxide nanoparticles have become key materials in biosensor design due to their superparamagnetic magnetic properties, chemical stability, biocompatibility, and low cost [183,184]. They can act as nanozymes, catalyzing peroxidase-like redox reactions for electrochemical and colorimetric detection of biomolecules (e.g., glucose, dopamine, nucleic acids) with nanomolar detection limits and high sensitivity [138,185,186,187].

In a brief parallel, with nanoparticles intensively used in nanotechnological processes and used extensively in numerous applications, gold nanoparticles are renowned for their plasmonic properties and the ability to immobilize biomolecules, offering high optical and electrochemical sensitivity. However, they present economic disadvantages and require stabilizers to prevent aggregation [188,189]. In this regard, iron oxide nanoparticles support magnetic manipulation, rapid separation from complex samples and easy integration into biosensors, all while maintaining low cost and excellent versatility [190,191].

From another perspective, a combined, hybrid approach of Fe/Au nanoparticles is emerging as a synergy with extremely promising potential. For instance, Fe/Au hybrids, obtained in the form of core–shell nanoparticles or dumbbell structures, represent a composite material with complementary functionalities, the magnetism of the iron oxide core and the plasmonic properties of gold, opening the way to complex applications in biosensors and theranostics [192,193,194]. A specific example in this case, in the core–shell structure, the Fe_3_O_4_ core contributes T_2_ contrast in magnetic resonance imaging and can supports heating (by magnetic hyperthermia) in alternating fields, and in the same time the outer gold layer locally enhances absorption at near-infrared (NIR) wavelengths and enables surface enhanced Raman spectroscopy (SERS), computed tomography (CT) imaging and photoacoustics [195,196].

The approach in which iron oxide is combined with other types of nanoparticles, such as silver, copper, platinum, ITO, or carbonaceous materials, has demonstrated remarkable potential in the development of biosensors with superior performance. Each of these combinations offers a specific set of functional properties that complement each other, leading to multifunctional systems. For instance, the Fe/Ag combination capitalizes on the bactericidal properties and high conductivity of silver, complementing the magnetic separation capacity and peroxidase activity of iron oxide, being effective in the detection of biological analytes under contaminated or complex conditions [158,159,167,190].

In the case of Fe/Cu, the redox catalytic properties of copper and its low cost recommend it as a complementary material in electrochemical biosensors intended for monitoring in accessible environments or for portable applications [172,173].

In contrast, the Fe/Pt hybrid structures offer catalytic efficiency in redox reactions and increased stability under varied pH and temperature conditions, being suitable for applications in the detection of glucose, hydrogen peroxide or other critical biomarkers [174,176]. Similarly, the integration of Fe_3_O_4_ with carbonaceous materials such as graphene or carbon nanotubes provides an extended active surface area and excellent conductivity, facilitating efficient electron transfer and amplification of the detection signal [177,178]. These hybrid platforms are particularly suitable for point-of-care applications, real-time monitoring, and integration into wearable or implantable devices (Table 7).

Depending on, but also in close correlation with, the specific application in which they are intended to be used, the diversity of iron oxide nanoparticles includes controlled synthetic variants (magnetite, maghemite, doped oxides and IONPs with different characteristics), aspects that significantly influence catalytic activity and magnetic properties [86,183,197].

In the design of biosensors for use in complex matrices such as serum, blood or wastewater, the main challenge is represented by biofouling, namely the nonspecific adsorption of proteins, polysaccharides and lipids that reduce the active surface area of the electrode and alter the analytical signal. This problem affects the sensitivity, selectivity and long-term stability, which is why antifouling strategies are essential in the development of hybrid platforms based on magnetic nanoparticles. Antifouling polymeric interfaces, especially those based on zwitterionic polymers or polyethylene glycol, can form dense hydrated layers that limit nonspecific interactions and maintain stability in complex biological environments [198,199]. An important role is played by natural membranes used as a protective layer and anchoring platform for bioreceptors. Chitosan, for example, is frequently used due to its biocompatibility, moderate conductivity and its ability to form stable films that reduce nonspecific adsorption and facilitate the immobilization of functional biomolecules [200,201]. Similarly, proteins such as albumin can be used to passivate surfaces, forming a barrier layer that limits the access of interfering biomolecules, but allows subsequent selective functionalization with antibodies or aptamers [202,203]. Another relevant example is model phospholipid membranes, which mimic the structure of the cellular bilayer and act both as an antifouling barrier and as a favorable environment for the anchoring of biomolecules. These structures allow the preservation of selective permeability for small molecules and can be regenerated after repeated exposure to complex samples, which gives them a high potential for applications in implanted or reusable biosensors [204,205].

A natural, sustainable, biocompatible alternative, and at the same time a great scientific breakthrough, are magnetosomes (Fe_3_O_4_ nanoparticles naturally produced by magnetotactic bacteria), wrapped in specific lipid membranes and arranged in uniform magnetic chains [109]. These magnetosomes offer perfectly controlled crystallinity, chemical purity, biocompatibility and very well-defined anisotropic magnetic properties. Moreover, the biological coating facilitates functionalization with enzymes, nucleic acids or antibodies and allows for directed magnetic manipulation, conferring stability in biological environments and efficiency in catalyzing redox reactions, without external additives [37,111,206].

Operational stability and reusability are essential criteria for evaluating the performance of nanozymes, including those derived from magnetotactic bacteria. In the case of magnetosomes, recent studies have demonstrated that these structures can maintain catalytic activity for extended periods, even under variable environmental conditions, due to the protection provided by the biological membrane and the high crystallinity of Fe_3_O_4_. This behavior contrasts with that of synthetic nanoparticles, which can rapidly lose their catalytic properties through aggregation or surface transformations. For example, a detailed study on the stability of superparamagnetic iron oxide nanoparticles showed that pH has a direct effect on the dispersion and available active surface area, and aggregation becomes significant under acidic conditions, which limits catalytic performance in various types of applications [207]. In comparison, magnetosomes maintain better stability to pH variations, which makes them more robust in biological and industrial contexts.

Another key element is catalytic performance. Synthetic Fe_3_O_4_-based nanozymes have been described as having significant peroxidase activities, but it has been shown that much higher catalytic constants can be achieved. Thus, in the case of an artificial nanozyme with peroxidase activity, bimolecular reaction constants up to 100 times higher than those of natural enzymes were obtained, demonstrating the extraordinary potential of these systems to outperform biological biocatalysts [208]. While these results highlight the advantages of optimized synthetic nanozymes, magnetosomes offer a unique combination of catalytic activity, stability and possibility of biological functionalization, which differentiates them from completely artificial analogues. However, still under study and being natural products, bacterial magnetite faces challenges, especially related to large-scale production that requires controlled biological conditions and rigorous purification, which increases costs [23,111,209]. From a practical perspective, the use of magnetosomes implies strict requirements regarding the identity, purity and safety of the materials. In particular, the control of endotoxin levels and batch reproducibility are mandatory conditions for validating the application in biomedical environments. In addition, a complete characterization of the catalytic properties, including the determination of kinetic parameters (Km and kcat) is necessary to demonstrate whether MTB-derived nanosomes can match or even surpass the performance of synthetic ones. Current data suggest that these particles can offer a superior balance between stability, reusability and catalytic efficiency, making them competitive candidates for biomedical and environmental applications, especially in scenarios where robustness and functional control are as important as maximum catalytic activity. For instance, trypsin solution was used either for delivering through an AFM nanopipette to locally digest dried albumin layers [210,211], or more recently, for immobilization on Fe_3_O_4_ at an optimal pH of 8, with a deep understanding of the events taking place at the nanoparticle-enzyme interface [212]. Nevertheless, it can be admitted, their biocompatibility, intrinsic catalytic activity and chain structure can allow the development of new generation nanosensors, characterized by sustainability, stability, precision and magnetic and multimodal manipulation capacity.

## 8. Conclusions

Iron oxide nanoparticles continue to represent a versatile and promising platform for the development of advanced biosensors, thanks to their magnetic properties, high biocompatibility and the ability to act as nanozymes in essential catalytic processes. While synthetic variants, including those obtained by ecological methods (green synthesis), offer the advantage of dimensional and morphological control, they can suffer from catalytic variability and limited stability under complex biological conditions.

In this regard, the authors are interested in what other sources of iron oxide exist. Are there any biocompatible, naturally synthesized alternatives? And if so, how do they compare to synthetic variants, and what are their qualities? This led us to analyze iron oxide nanoparticles biomineralized by magnetotactic bacteria in the form of magnetosomes, as they represent a natural and ultra-specialized alternative, with a perfectly ordered crystalline structure, a uniform dimensional dispersion, and a biological coating that confers them both stability in physiological environments and a superior potential for biomolecular functionalization. Although the production of these nanoparticles involves greater technological and logistical constraints, their unique properties, including inherent nanoenzymatic activity, targeted magnetic manipulability, and excellent biocompatibility, recommend them as key elements for the next generation of smart nanosensors capable of multimodal detection and therapeutic integration.

The comparison between synthetic and bacterial nanoparticles highlights an important transition from artificial systems, constructed with a high degree of control, to biologically inspired systems, optimized by evolution and directly adapted to interact with biological environments. Therefore, the integration of iron nanoparticles into biosensors is no longer just a technological solution, but a fundamental pillar of emerging biotechnologies. To the authors’ knowledge, this paper highlights for the first time the possibility of using natural iron nanoparticles, biomineralized by magnetotactic bacteria, in the construction of state-of-the-art nanobiosensors capable of overcoming the fundamental limitations of synthetic nanoparticles, offering a sustainable, accurate, and scalable alternative for advanced biomedical applications.

## Figures and Tables

**Table 1:** Main characteristics underlying the advantages and disadvantages of natural enzymes vs. nanozymes.

Characteristic	Natural Enzymes	Nanozymes	**Ref.**
composition	proteins, composed of amino acid chains folded into specific three-dimensional structures	nanomaterials with enzyme-mimicking catalytic properties: - metal nanoparticles; - metal oxides; - carbon-based materials; - metal organic frameworks	[45]
specificity	- very high specificity; - active site highly selective	generally lower specificity can be improved through surface functionalization and ligand attachment	[46]
stability	sensitive to environmental factors: - prone to denaturation under extreme pH, temperature, or presence of proteases	high stability under harsh conditions: - including extreme pH and temperature; - resistant to proteolytic degradation	[47]
catalytic activity	- high specificity and efficiency under physiological conditions; - often limited to narrow pH and temperature ranges	- tunable activity, can function under a broader range of conditions; - activity can be enhanced through structural and surface modifications	[48]
production cost	- high, requires complex expression systems; - expensive production, purification, and storage conditions	- lower, synthesized through relatively simple chemical methods; - scalable production	[49]
reusability	- limited - often lose activity after a single use or over time	high, can retain activity over multiple cycles without activity loss	[50]
bio- compatibility	generally safe and non-toxic	varies, some nanozymes may exhibit cytotoxicity depending on composition and size, surface modifications can enhance biocompatibility	[45]
functional diversity	- limited to native biological roles, engineering is complex	broad, can be engineered to mimic various enzymatic activities (e.g., peroxidase, oxidase, catalase), multifunctional capabilities	[51]
environmental senstivity	- highly sensitive, activity can be affected by slight changes in environmental conditions	robust; maintain activity under a wide range of environmental conditions	[52]
storage and shelf-life	require specific storage conditions (e.g., low temperatures), limited shelf-life	stable at room temperature, longer shelf-life, easier storage and transportation	[53]
toxicity concerns	- minimal; - generally recognized as safe	- potential toxicity depending on material composition and accumulation; - requires thorough biocompatibility assessments	[45]
application scope	- widely used in biological systems, diagnostics, and therapeutics; - limitations in industrial applications due to stability issues	expanding applications in: - biosensing; - environmental remediation; - therapeutics, medicine; - industrial catalysis	[54]
design flexibility	- limited; - modifications can affect activity and stability	high, properties can be tailored (size, shape, composition, and surface modifications to suit specific applications)	[55]
regulatory approval	- many are approved for clinical use; - well-understood mechanisms;	- emerging field; - regulatory pathways are still being Established; - requires comprehensive safety and efficacy evaluations	[54]
scalability	- challenging; - complex production processes limit large-scale manufacturing	- highly scalable, chemical synthesis methods allow for mass production	[56]
variability	low—sequence-defined, reproducible	moderate to high—adjustable via engineering	[57,58]
environmental footprint	biodegradable, minimal environmental impact	depends on composition, some nanozymes may persist	[59]
clinical suitability	high, generally safe	variable, depends on toxicity and surface functionalization	[60]

**Table 2:** Comparison of iron oxide nanoparticle synthesis methods.

Method	Advantages	Limitations	Ref.
co-precipitation	simple, cost-effective, high yield, scalable	- broad particle size distribution, - low crystallinity, aggregation	[90]
thermal decomposition	- excellent size and shape control, - high crystallinity	- high temperature, - toxic solvents, - expensive precursors	[91]
microemulsion	- fine control over particle size - shape, surfactant-stabilized	- expensive surfactants, - purification required, - poor scalability	[92]
sol-gel	- uniform particle size, - good chemical homogeneity, - low temperature process	- time-intensive, - solvent residues, - not always scalable	[91]
laser ablation	- high purity, - no chemical contamination, - precise control	- expensive equipment, - low yield, - not suitable for large-scale production	[90]
biological/ green synthesis	- environmentally friendly, - biocompatible particles, - mild reaction conditions	- limited reproducibility, - poor control over size and shape, - lower crystallinity	[93]

**Table 3:** Main characterization techniques for iron oxide nanoparticles.

Technique	Main Parameters Assessed	Significance	Ref.
TEM	- particle size - morphology	- high-resolution imaging of shape - size distribution	[91,94]
SEM	- surface morphology - agglomeration	- analysis of topography - particle clustering	[91,94]
XRD	- crystal structure	- differentiates magnetite/maghemite, - estimates crystallinity	[95,96]
FTIR	- surface functional groups, - bonding interactions	- detection of organic coating agents, - surface coatings - bioconjugation	[97]
DLS	- hydrodynamic diameter, - polydispersity index	- size analysis in colloidal systems, - evaluates nanoparticle aggregation	[98]

**Table 4:** Sources of iron oxide nanoparticles.

Method Source	Description	Advantages	Limitations	Variability	Environmental Footprint	Clinical Suitability	Ref.
co-precipitation	- reaction between Fe^2+^ and Fe^3+^ salts in an alkaline medium	- simple, -cost-effective high yield	- limited control over size and shape	moderate	low, uses common salts, minimal waste	moderate, generally safe but requires purification for biomedical use	[102]
thermal decomposition	- high-temperature decomposition of iron organometallic precursors in solvents	- precise control of ize and monodispersity	- expensive, - requires specialized equipment	low	moderate, high energy consumption, organic solvents may be toxic	high, produces uniform nanoparticles suitable for clinical applications after proper surface modification	[90]
continuous flow synthesis	- scalable synthesis with controlled parameters (pH, temperature, flow rate)	- excellent size control, - suitable for industrial scaling	- complex system	low	moderate, energy consumption	high, scalable method compatible with biomedical applications after functionalization	[99]
physical methods	- methods like evaporation– condensation, laser ablation	- high purity	- low yield, - high cost	low	moderate, energy-intensive, solvent-free methods reduce chemical waste	moderate, nanoparticles may need surface modification for biocompatibility	[90]
green synthesis	- use of plant extracts or microorganisms for reduction of iron ions	- increased biocompatibility	- low reproducibility, - less control over nanoparticle properties	high	low, eco-friendly, uses renewable materials, minimal toxic byproducts	high, inherently biocompatible, ideal for biomedical applications	[100,101]
alternative sources	- use of iron-containing waste in chemical or electrochemical methods	- sustainable, -low-cost	- variable purity, - possible contamination	high	low to moderate, promotes recycling but potential contamination	moderate, requires purification for safe biomedical use	[103]

**Table 5:** Optimal conditions and kinetic parameters for IONP and magnetosomes activities.

Nanozymes (Similar Sizes)	Substrate	Km (mM)	Vmax (M s^−1^)	Kcat (s^−1^)	pH	Temperature (°C)	Mimicking Activity	Ref.
Fe_3_O_4_ (44 nm) synthetic	H_2_O_2_	54.6	1.8×10^−8^		7.4	30	Peroxidase	[122]
	TMB	0.374	2.6×10^−8^					
Fe_3_O_4_ (55 nm) synthetic	ABTS	0.12–0.96	0.52–6.10 × 10^−7^	0.25–2.9 × 10^−4^		RT (20–25 °C)	Peroxidase	[122]
	TMB	0.24–0.71	0.42–2.4 × 10^−7^	0.2–1.14 × 10^−4^		RT (20–25 °C)	Peroxidase	
Fe_3_O_4_ (52 nm) magnetosome	H_2_O_2_	170.65	9.33 × 10^−9^		4	28	Peroxidase	[122]
	TMB	0.90	4.45 × 10^−9^					
Fe_3_O_4_ (40–45 nm) magnetosome (e.g., MSR-1)	TMB	1.215	8.06 × 10^−8^		4–6	40–60	Peroxidase	[125]
	H_2_O_2_	100.3	3.7 × 10^−8^					

**Table 6:** Iron oxide nanoparticles-based biosensors vs. enzymatic biosensors.

Characteristics	Iron Oxide NP Based Biosensors	Conventional Enzymatic Biosensors	Ref.
sensitivity	- high, enables the detection of microRNAs via magnetically induced electrochemical amplification	- sensitive, but activity may fluctuate due to enzyme degradation in complex matrices and low analyte concentrations	[131,132]
specificity	- requires surface functionalization with aptamers, antibodies, or ligands potential for non-specific interactions	- high substrate specificity due to natural enzyme-substrate recognition	[133,134]
preconcentration capability	- enables magnetic preconcentration of analytes, enhance active surface area and signal-to-noise ratio	- lack inherent preconcentration, - signal depends entirely on substrate diffusion and enzymatic turnover	[135]
stability and durability	- stability across varying pH, temperature and environmental conditions, - magnetic separation allows reuse	- limited by enzyme denaturation, pH/temperature sensitivity, and short shelf-life	[136]
fabrication complexity	- synthesis involves multistep nanoparticle production and surface modifications, - requires precision in size, - dispersion, - chemical coating	- established immobilization methods (adsorption, entrapment) - require careful optimization for maximal enzyme activity	[136]
portability	- well-suited for integration into portable platforms	- commercial enzymatic devices exist (e.g., glucose test strips), - enzyme instability can limit field lifespan	[90]
biocompatibility	- generally biocompatible, - high concentrations may induce ROS and cytotoxic effects	- biodegradable enzymes pose minimal risk, - rare allergenic responses	[90]
cost and scalability	- cost-efficient and scalable synthesis, - including green chemistry routes	- enzyme purification - cold-chain logistics increase costs	[137]
limit of detection (LOD)	0.60–0.90 ppb	- nanomolar (nM) to micromolar (µM) range, depending on the type of enzyme and the electrochemical or optical signaling method used	[138,139]
range	10–100 ppb	a few nM/µM to hundreds of µM	[138,139]
response time	<30 s	a few seconds to a few minutes	[140,141,142]
regeneration cycles	7	varies, some require active regeneration	[143,144]
sample matrix	various applications in complex environments (blood, serum, urine, environmental water, food samples)	applications in complex environments, enzyme stability may be affected (blood, plasma, urine, saliva, food extracts, buffer solutions)	[140,145]

**Table 7:** Analytical performance of hybrid platforms based on iron oxide nanoparticles. For the studies below, the LOD was not reported.

Hybrid NP Systems	Composition	Detection Mode	Target Analyte	Enhancement Mechanism	Ref.
Au-Fe_3_O_4_	Fe_3_O_4_ + Au (core–shell, composites)	colorimetric, electrochemical, SERS	glucose, H_2_O_2_, biomolecules	synergistic peroxidase-like catalysis, plasmonic amplification, magnetic separation	[154,185,189]
Ag-Fe_3_O_4_	Fe_3_O_4_ + Ag (core–shell, hydrogel, composites)	optical (plasmonic, SERS)	various bacteria, contaminants	bactericidal effect, SERS amplification, magnetic control	[157,159,164,165]
Cu-Fe_3_O_4_	Fe_3_O_4_ doped with CuO/Cu_2_O, composites	electrochemical, optical	glucose, urea	electrocatalysis, magnetic separation	[170,171,172,173]
Pt-Fe_3_O_4_	Fe_3_O_4_ + Pt (thin layer, core–shell)	electrochemical, colorimetric	glucose, H_2_O_2_, nitrite, other redox analytes	synergistic peroxidase-like activity, strong Pt electrocatalysis, magnetic recovery of sensor	[174,176]
C-Fe_3_O_4_	Fe_3_O_4_ + Graphene/CNTs/ amorphous carbon	electrochemical, colorimetric	Pb^2+^, H_2_O_2_, glucose	conductivity, peroxidase-like, rapid immobilization	[177,178,179,180]
ITO-Fe_3_O_4_	Fe_3_O_4_ immobilized on ITO electrodes	electrochemical, optoelectronic	biomarkers, enzymes, H_2_O_2_	synergy of Fe_3_O_4_ (magnetic immobilization and peroxidase) with ITO (conductivity and transparency)	[181,182]

## Data Availability

Data sharing is not applicable.
